# Development and validation of nomogram including high altitude as a risk factor for COPD: A cross-sectional study based on Gansu population

**DOI:** 10.3389/fpubh.2023.1127566

**Published:** 2023-03-02

**Authors:** Ao Lin, Chun Mao, Boqi Rao, Hongjun Zhao, Yunchao Wang, Guokang Yang, Haisheng Lei, Chenli Xie, Dongsheng Huang, Yibin Deng, Xuhui Zhang, Xinhua Wang, Jiachun Lu

**Affiliations:** ^1^The State Key Lab of Respiratory Disease, The First Affiliated Hospital, Institute of Public Health, Guangzhou Medical University, Guangzhou, China; ^2^Department of Pulmonary and Critical Care Medicine, Quzhou People's Hospital, The Quzhou Affiliated Hospital of Wenzhou Medical University, Quzhou, China; ^3^Institute of Basic Medicine, Institute of Public Health, Gansu University of Chinese Medicine, Lanzhou, China; ^4^Department of Respiratory and Critical Care Medicine, Dongguan Binwan Central Hospital, Dongguan, China; ^5^Department of Respiratory and Critical Care Medicine, Shenzhen Longhua District Central Hospital, Shenzhen, China; ^6^Centre for Medical Laboratory Science, The Affiliated Hospital of Youjiang Medical University for Nationalities, Baise, China; ^7^Department of Respiratory Medicine, The Affiliated Hospital of Gansu University of Chinese Medicine, Lanzhou, China

**Keywords:** COPD, altitude, prevalence, risk factor, nomogram

## Abstract

**Background:**

Chronic Obstructive Pulmonary Disease (COPD) is a common and harmful disease that requires an effective tool to early screen high-risk individuals. Gansu has unique environments and customs, leading to the different prevalence and etiology of COPD from other regions. The association between altitude and COPD once attracted epidemiologists' attention. However, the prevalence in Gansu and the role of altitude are still unclarified.

**Methods:**

In Gansu, a multistage stratified cluster sampling procedure was utilized to select a representative sample aged 40 years or older. The questionnaire and spirometry examination were implemented to collect participants' information. The diagnosis and assessment of COPD were identified by the Global Initiative for Chronic Obstructive Lung Disease (GOLD) criterion, while post-bronchodilator FEV_1_/FVC < LLN was for sensitivity analysis. Furthermore, the effect of high altitude on COPD was evaluated by the logistic regression model after propensity score matching (PSM). Finally, the participants were randomly divided into training and validation sets. The training set was used to screen the relative factors and construct a nomogram which was further assessed by the receiver operating characteristic (ROC) curve, calibration curve, and decision curve analysis (DCA) in the two sets.

**Results:**

There were 2,486 eligible participants in the final analysis, of which 1,584 lived in low altitudes and 902 lived in high altitudes. Based on the GOLD criterion, the crude and standardized prevalences in Gansu were 20.4% (18.7–22.0) and 19.7% (17.9–21.6). After PSM, the logistic regression model indicated that high altitude increased COPD risk [PSM OR: 1.516 (1.162–1.978)]. Altitude, age, sex, history of tuberculosis, coal as fuel, and smoking status were reserved for developing a nomogram that demonstrated excellent discrimination, calibration, and clinical benefit in the two sets.

**Conclusions:**

COPD has become a serious public health problem in Gansu. High altitude is a risk factor for COPD. The nomogram has satisfactory efficiency in screening high-risk individuals.

## Introduction

Chronic Obstructive Pulmonary Disease (COPD) is the most common chronic respiratory disease and is the third cause of mortality leading to 3.2 million deaths worldwide (1, 2). A population-based survey conducted from 2002 to 2004 indicated that the prevalence of Chinese older than 40 was about 8.2% (3). The rate rose to 13.6% just a decade later, which was higher in men and the southwest region than in women and other regions (4). Airway limitation and respiratory symptoms would seriously disturb the patient's life and work due to the irreversible and progressive lung lesions (5). Besides, data demonstrated that the direct medical cost of COPD accounted for 33.33%−118.09% of the average annual income in China (6). COPD has increasingly become a serious public health problem in China, leading to tremendous economic, social, and healthcare burdens.

Gansu province, located in northwestern China, is characterized by various altitudes (900–3,800 m) and multi-ethnic habitation (7, 8). Compared to the flat region, the high-altitude region has heterogeneity in the prevalence and etiology of many diseases due to particular environments (cold temperatures, low humidity, hypobaric, hypoxic, etc.) and lifestyles (coal or biomass as fuel, etc.) (9–11). But, the prevalence of COPD in Gansu is still unclarified. In addition, COPD is mediated by environmental and genetic factors, in which smoking is the most recognized risk factor (2). However, quite a few non-smokers developing COPD indicate its etiological complexity (12). Although some researchers advocated that altitude was associated with COPD, it has yet to reach a consensus (13–15).

In this study, we conducted a cross-sectional study to explore the prevalence of COPD in Gansu and clarify the association between altitude and COPD. Also, a nomogram enrolling candidate risk factors was constructed to screen the high-risk population.

## Methods

### Sampling and participants

Based on Equations 1, 2, we took deff = 5, α = 95%, *p* = 10.2%, *r* = 20%, and response rate = 90% to calculate the sample size. The referenced prevalence was derived from previous research (4).


(1) $$\begin{array}{l} N=deff\frac{\mu_{\alpha }^{2}p\left(1-p\right)}{d^{2}} \end{array}$$

(2) $$\begin{array}{l} d=pr \end{array}$$

**Figure 1:**
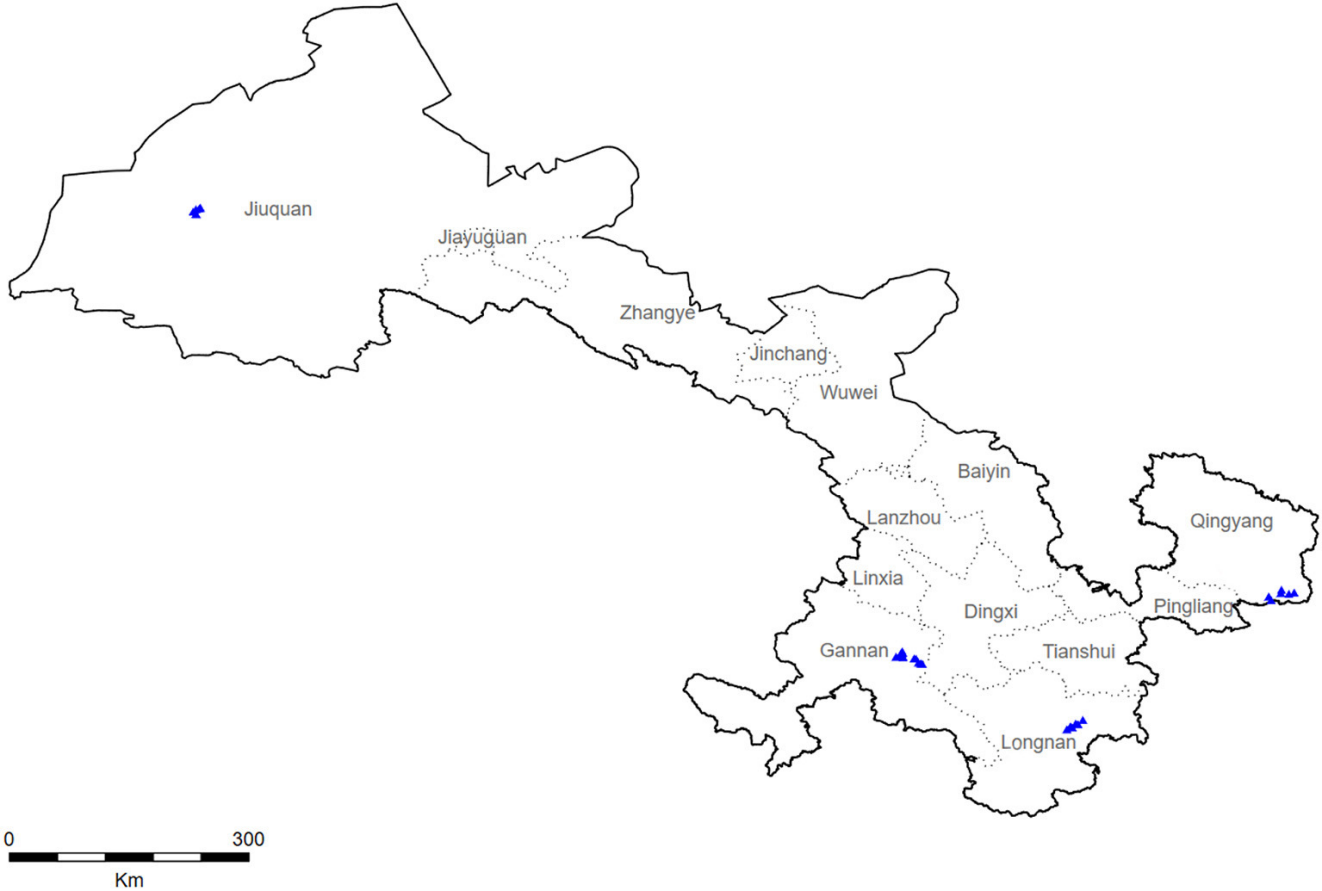
Survey points in Gansu Province including Jiuquan, Gannan, Longnan, and Qingyang.

During 2018–2019, a multistage stratified cluster sampling procedure was utilized to collect a representative sample from Gansu (Figure 1). Specifically, four monitoring sites in Gansu were selected by a probability proportional to size method (Longnan City, Jiuquan City, Qingyang City, and Gannan City). Using the same method, three towns were selected from each city, and more than two villages were further selected from each town. Subsequently, we selected a village with more than 150 households by the cluster random sampling method and randomly selected 100 families with members older than 40. Finally, the KISH table was used to select an eligible member from each family for investigation.

**Figure 2:**
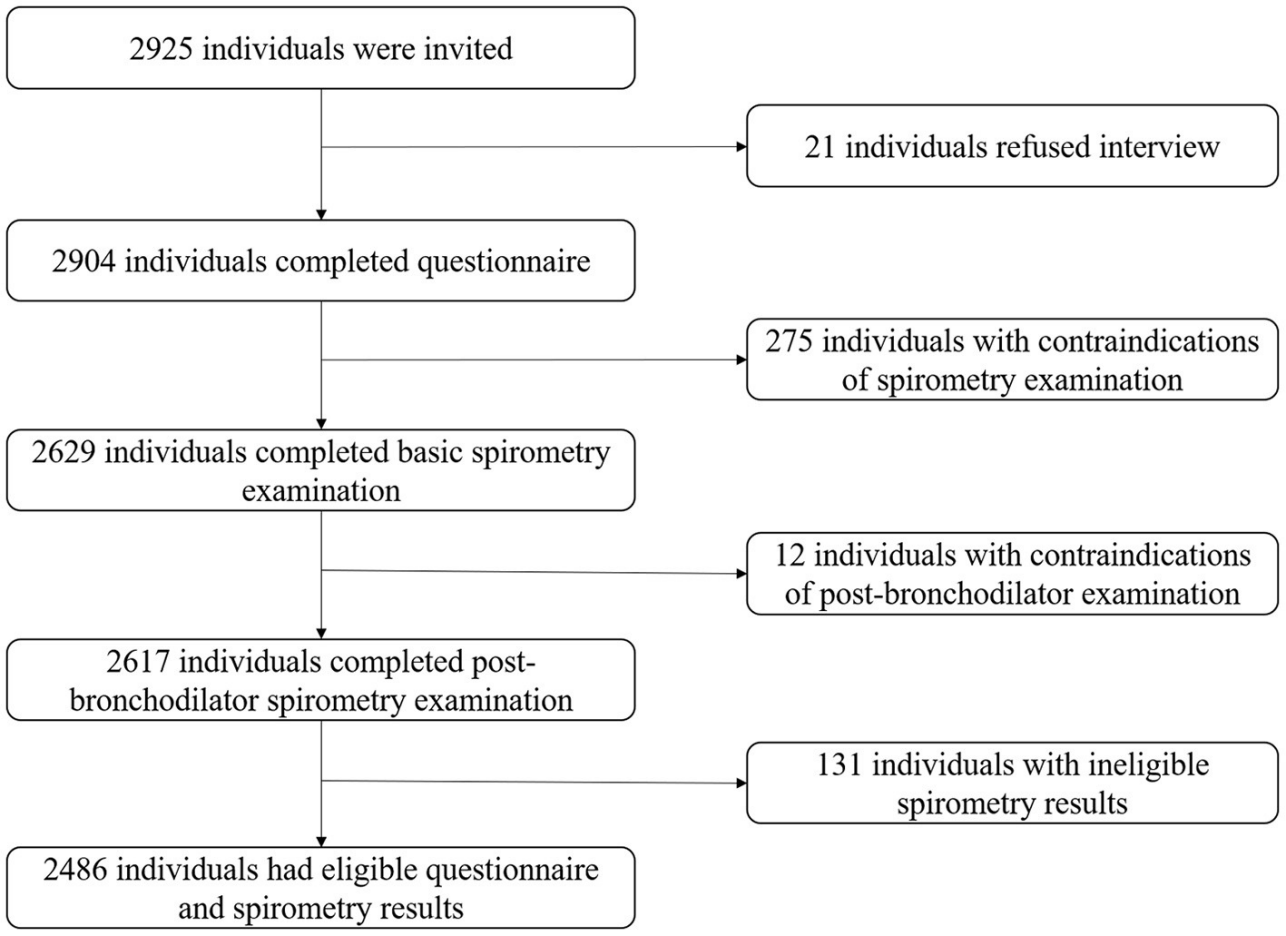
Flowchart of screening participants.

All participants were Chinese residents older than 40. Those physically unable to complete the spirometry examination were excluded (active tuberculosis, pregnancy, cardiovascular and cerebrovascular accidents in the past month, heart rate >120 beats/min or blood pressure >180/120 mmHg, related surgeries in the past 3 months, etc.) (16). The flowchart of screening participants was exhibited in Figure 2. Our study was approved by the ethics review committee of Guangzhou Medical University and Xi'an Jiaotong University Health Science Center.

### Procedures

A professional questionnaire was implemented to collect information about demographic characteristics, respiratory symptoms, and exposure to COPD-related risk factors (17). Specifically, the smokers were defined as those who had continually smoked for more than 6 months. The former smokers were defined as those who had quit smoking for more than 1 month when they were interviewed. Biomass was defined as wood, grass, animal dung, and crop waste. Coal was defined as coal and lignite. Occupational exposure was defined as exposure to dust or chemicals in the workplace for more than 1 year (4, 17). According to some references, the physiological alteration of the respiratory and circulatory systems began at altitudes >1,500 m (18–21). Thus, we chose 1,500 m to divide low and high altitudes.

Lung function was examined by EasyOne Spirometer (NDD Medizintechnik AG, Switzerland) following the operation of the American Thoracic Society and the European Respiratory Society (16). In brief, eligible participants were asked to estimate basic lung function and rechecked 15 min after inhalation of 400 μg salbutamol. Each test was acceptable only when the quality was rated A, B, or C. The overall proportion of A should be more than 70%.

The questionnaire and the spirometry examination were performed by trained staff from local medical systems.

### Outcomes

According to the Global Initiative for Chronic Obstructive Lung Disease (GOLD) 2019, participants were diagnosed with COPD if forced expiratory volume in one second (FEV_1_): forced vital capacity (FVC) was <70% after inhaling bronchodilators. The patients were categorized as the GOLD stage I (mild group: FEV_1_ ≥80% predicted), GOLD stage II (moderate group: FEV_1_ ≥50 to <80% predicted), GOLD stage III (severe group: FEV_1_ ≥30 to <50% predicted), and GOLD stage IV (very severe group: FEV_1_ <30% predicted). The modified Medical Research Council (mMRC) dyspnea scale was used to estimate the dyspnea of COPD patients (5).

In this study, another diagnostic criterion was available for sensitivity analysis: post-bronchodilator FEV_1_/FVC < the lower normal limit (LLN). The LLN, predicted FEV_1_ and FVC were calculated by specialized formulas that considered the characteristics of the Chinese population (22).

### Statistical analysis

Continuous variables were described by mean [standard deviation (SD)] or median (lower quartile, upper quartile) and were compared by *T*-test or Mann–Whitney test. Categorical variables were described by rate [95% confidence interval (95% CI)] and were compared by Chi-square test or Fisher's exact test. The Cochran-Armitage test was used to compare the difference between two groups of ordinal data. The overall standardized prevalence of COPD was calculated based on the sample structure of the 2010 census of the Chinese population (23).

The 1:1 propensity score matching (PSM), with the caliper of 0.02 and in the nearest method, was implemented to balance the populations' characteristics between low and high altitudes. Subsequently, logistic regression models estimated odd ratios (Ors) and 95% CI to evaluate the association between high altitude and COPD risk (24).

Furthermore, the participants were randomly divided into the training and validation sets at the ratio of 6:4. In the training set, the univariable and stepwise logistic regression models were performed to screen the related factors to construct a nomogram for COPD (GOLD criterion). The nomogram was assessed by the receiver operating characteristic (ROC) curve, calibration curve, and decision curve analysis (DCA), which was reassessed in the validation set for internal validation.

Statistical analysis was performed by SPSS 26.0 and R 4.13. The result was considered statistically significant only when the two-sided *P* was < 0.05.

## Result

### Participants characteristics

We interviewed 2,925 residents. Finally, 2,486 eligible participants were included in the analysis, of which 1,584 lived in low altitudes (mean: 1,190.56 m, range: 996–1,442 m) and 902 lived in high altitudes (mean: 2,684.92 m, range: 2,416–2,896 m). Compared to the low-altitude population, the high-altitude population showed significant differences in demographic characteristics except for body mass index (BMI), history of tuberculosis (TB), and coal as fuel (*P* < 0.05; Table 1).

**Table 1:** Demographic characteristics and exposures of Gansu participants aged 40 years or older.

**Variables**	**Total (*n* = 2,486)**	**Low altitudes (*n* = 1,584)**	**High altitudes (*n* = 902)**	***P*-value^d^**
Altitude (m)^a^	1,732.76 (996, 2,896)	1,190.56 (996, 1442)	2,684.92 (2,416, 2,896)	**< 0.001**
Age (years)^b^	54.93 ± 9.33	54.12 ± 9.13	56.35 ± 9.52	**< 0.001**
BMI (kg/m^2^)^b^	24.37 ± 3.48	24.46 ± 3.55	24.22 ± 3.35	0.098
**Sex**				
Female	1,166 (46.9%)	682 (43.1%)	484 (53.7%)	**< 0.001**
Male	1,320 (53.1%)	902 (56.9%)	418 (46.3%)	
**Ethnic groups**				
Han	1,890 (76.0%)	1,568 (99.0%)	322 (35.7%)	**< 0.001**
Others	596 (24.0%)	16 (1.0%)	580 (64.3%)	
**Childhood hospital admission for severe respiratory disease**				
No	2,327 (93.6%)	1,532 (96.7%)	795 (88.1%)	**< 0.001**
Yes	159 (6.4%)	52 (3.3%)	107 (11.9%)	
**History of tuberculosis**				
No	2,468 (99.3%)	1,576 (99.5%)	892 (98.9%)	0.088
Yes	18 (0.7%)	8 (0.5%)	10 (1.1%)	
**Educational level**				
Primary school or below	1,394 (56.1%)	827 (52.2%)	567 (62.9%)	**< 0.001**
Middle or high school	1,021 (41.1%)	716 (45.2%)	305 (33.8%)	
College or above	71 (2.9%)	41 (2.6%)	30 (3.3%)	
**Coal as fuel**				
No	1,151 (46.3%)	754 (47.6%)	397 (44.0%)	0.085
Yes	1,335 (53.7%)	830 (52.4%)	505 (56.0%)	
**Biomass as fuel**				
No	400 (16.1%)	393 (24.8%)	7 (0.8%)	**< 0.001**
Yes	2,086 (83.9%)	1,191 (75.2%)	895 (99.2%)	
**Smoking status**				
Never	1,588 (63.9%)	916 (57.8%)	672 (74.5%)	**< 0.001**
Ever	154 (6.2%)	130 (8.2%)	24 (2.7%)	
Now	744 (29.9%)	538 (34.0%)	206 (22.8%)	
**Occupational exposure**				
No	1,516 (61.0%)	715 (45.1%)	801 (88.8%)	**< 0.001**
Yes	970 (39.0%)	869 (54.9%)	101 (11.2%)	
Pre-FEV_1_ (L)^c^	2.60 (2.20, 3.10)	2.70 (2.30, 3.30)	2.40 (2.00, 2.90)	**< 0.001**
Pre-FVC (L)^c^	3.50 (3.00 4.30)	3.80 (3.10, 4.50)	3.20 (2.80, 3.90)	**< 0.001**
Pre-FEV_1_/FVC (%)^c^	75.00 (69.00, 79.60)	75.40 (69.70, 80.05)	74.10 (68.20, 78.70)	**< 0.001**
Pre-FEV_1_ % predicted^c, e^	95.63 (84.50, 107.45)	98.38 (87.43, 111.05)	90.90 (80.27, 101.62)	**< 0.001**
Pre-FVC % predicted^c, e^	107.16 (96.03, 119.54)	110.62 (98.37, 122.20)	102.67 (92.66, 112.74)	**< 0.001**

BMI, body-mass index; FVC, forced vital capacity; FEV_1_, forced expiratory volume in the first second.

a Mean (minimum, maximum).

b Mean (standard deviation, SD).

c Median (P_25_, P_75_).

d Low altitudes vs. high altitudes.

e Five patients older than 81 years were excluded.

Bold values denote significant *P* values (*P* < 0.05).

### The prevalence of COPD in Gansu province

There were 508 individuals diagnosed with COPD by post-bronchodilator FEV_1_/FVC < 70%. The overall and overall standardized prevalences were 20.4% (18.7–22.0) and 19.7% (17.9–21.6). Taking 1,500 m as the boundary to divide low and high altitudes, we found that the prevalence in high altitudes was significantly higher than in low altitudes [high altitudes vs. low altitudes: 23.4% (20.7–26.4) vs. 18.8% (16.9–20.5), *P* = 0.006]. This tendency was also statistically significant in some subgroups (*P* < 0.05). The details were summarized in Table 2.

**Table 2:** Prevalence of COPD in Gansu participants aged 40 years and older by different characteristics (GOLD criterion).

**Variables**	**Total**		**Low altitudes**		**High altitudes**		***P* ^a^**
	**Case/total**	**Prevalence (95%CI)**	**Case/total**	**Prevalence (95%CI)**	**Case/total**	**Prevalence (95%CI)**	
Overall	508/2,486	20.4% (18.7–22.0)	297/1,584	18.8% (16.9–20.5)	211/902	23.4% (20.7–26.4)	**0.006**
**Sex**							
Female	161/1,166	13.8% (11.8–15.8)	79/682	11.6% (9.3–14.0)	82/484	16.9% (13.6–20.4)	**0.009**
Male	347/1,320	26.3% (23.9–28.7)	218/902	24.2% (21.3–26.8)	129/418	30.9% (26.3–35.5)	**0.010**
*P*-value		**< 0.001**		**< 0.001**		**< 0.001**	
**Age (years)**							
40–50	86/824	10.4% (8.5–12.7)	67/576	11.6% (9.1–14.5)	19/248	7.7% (4.6–11.1)	0.087
50–60	150/870	17.2% (14.9–19.7)	93/543	17.1% (14.1–20.5)	57/327	17.4% (13.4–21.6)	0.908
60–70	191/592	32.3% (28.4–36.2)	103/365	28.2% (23.6–32.9)	88/227	38.8% (32.4–45.2)	**0.008**
≥70	81/200	40.5% (33.5–47.7)	34/100	34.0% (24.7–43.9)	47/100	47.0% (37.0–57.5)	0.061
*P*_trend_		**< 0.001**		**< 0.001**		**< 0.001**	
**Ethnic groups**							
Han	369/1,890	19.5% (17.7–21.2)	295/1,568	18.8% (16.9–20.5)	74/322	23.0% (18.8–27.8)	0.086
Others	139/596	23.3% (19.9–27.0)	2/16	12.5% (2.0–38.0)	137/580	23.6% (20.2–27.3)	0.460
*P*-value		**0.045**		0.748		0.828	
**Childhood hospital admission for severe respiratory disease**							
No	468/2,327	20.1% (18.3–21.8)	287/1,532	18.7% (16.7–20.5)	181/795	22.8% (19.9–25.8)	**0.021**
Yes	40/159	25.2% (18.3–31.8)	10/52	19.2% (9.2–30.2)	30/107	28.0% (19.5–37.4)	0.230
*P*-value		0.127		0.928		0.227	
**History of tuberculosis**							
No	500/2,468	20.3% (18.5–21.8)	294/1,576	18.7% (16.6–20.4)	206/892	23.1% (20.3–25.9)	**0.008**
Yes	8/18	44.4% (23.1–69.2)	3/8	37.5% (9.0–76.0)	5/10	50.0% (16.7–83.3)	0.596
*P*-value		**0.025**		0.364		0.105	
**Educational level**							
Primary School or Below	291/1,394	20.9% (18.7–23.1)	146/827	17.7% (15.1–20.3)	145/567	25.6% (21.9–29.5)	**< 0.001**
Middle or high school	208/1,021	20.4% (17.7–22.8)	147/716	20.5% (17.5–23.5)	61/305	20.0% (15.7–24.7)	0.847
College or above	9/71	12.7% (5.3–20.7)	4/41	9.8% (3.0–23.0)	5/30	16.7% (3.7–30.7)	0.615
*P*_trend_		0.295		0.529		**0.041**	
**Coal as fuel**							
No	219/1,151	19.0% (16.7–21.4)	133/754	17.6% (14.8–20.4)	86/397	21.7% (17.7–25.8)	0.098
Yes	289/1,335	21.6% (19.5–23.7)	164/830	19.8% (16.9–22.2)	125/505	24.8% (21.2–28.9)	**0.032**
*P*-value		0.106		0.280		0.276	
**Biomass as fuel**							
No	79/400	19.8% (15.8–23.8)	77/393	19.6% (15.6–23.7)	2/7	28.6% (4.0–71.0)	0.910
Yes	429/2,086	20.6% (18.8–22.3)	220/1,191	18.5% (16.2–20.6)	209/895	23.4% (20.6–26.4)	**0.006**
*P*-value		0.711		0.622		1.000	
**Smoking status**							
Never	266/1,588	16.8% (14.9–18.7)	131/916	14.3% (12.0–16.6)	135/672	20.1% (17.2–23.4)	**0.002**
Ever	30/154	19.5% (13.4–26.0)	25/130	19.2% (12.6–25.9)	5/24	20.8% (5.3–38.9)	1.000
Now	212/744	28.5% (25.2–31.8)	141/538	26.2% (22.3–29.9)	71/206	34.5% (28.1–41.6)	**0.026**
*P*_trend_		**< 0.001**		**< 0.001**		**< 0.001**	
**Occupational exposure**							
No	315/1,516	20.8% (18.7–23.0)	131/715	18.3% (15.5–21.0)	184/801	23.0% (20.0–26.1)	**0.026**
Yes	193/970	19.9% (17.4–22.2)	166/869	19.1% (16.5–21.7)	27/101	26.7% (18.4–35.5)	0.069
*P*-value		0.595		0.692		0.400	
**BMI**							
Normal	248/1,165	21.3% (19.0–23.6)	141/734	19.2% (16.5–22.0)	107/431	24.8% (20.8–28.9)	**0.024**
Overweight	172/904	19.0% (16.6–21.7)	101/575	17.6% (14.4–20.6)	71/329	21.6% (17.3–26.6)	0.139
Obesity	73/365	20.0% (15.9–24.4)	48/246	19.5% (14.5–24.5)	25/119	21.0% (14.2–28.8)	0.738
Malnutrition	15/52	28.8% (17.3–40.4)	7/29	24.1% (8.3–41.7)	8/23	34.8% (13.6–55.6)	0.400
*P*_trend_		0.861		0.894		0.707	

COPD, chronic obstructive pulmonary disease; BMI, body-mass index; CI, confidence interval.

a Low altitudes vs. high altitudes.

Bold values denote significant *P* values (*P* < 0.05).

There were 538 individuals diagnosed with COPD by post-bronchodilator FEV_1_/FVC < LLN. The overall and overall standardized prevalences were 21.7% (20.2–23.3) and 21.3% (19.5–23.3). The prevalence in high altitudes was significantly higher than in low altitudes [high altitudes vs. low altitudes: 24.7% (22.0–27.8) vs. 20.0% (18.0–22.1), *P* = 0.006]. This tendency was also statistically significant in some subgroups (*P* < 0.05). The details were summarized in Supplementary Table S1.

### The assessment of patients

The formula calculating the predicted FEV_1_ was suitable for the participants aged 40–81 years, so five patients older than 81 were excluded. Finally, 505 patients were included in the severity assessment. Among the eligible patients, the proportion of GOLD stage I, GOLD stage II, and GOLD stage III or IV was 67.1% (63.0–71.5), 28.7% (24.8–33.1), and 4.2% (2.6–5.9), respectively. The result of the mMRC dyspnea scale showed that the proportion of Grade 0, Grade 1, Grade 2, Grade 3, and Grade 4 was 55.1% (51.0–59.3), 17.3% (14.0–20.7), 16.7% (13.8–20.3), 5.3% (3.31–7.3), and 5.3% (3.31–7.3), respectively. Furthermore, compared to low-altitude patients, high-altitude patients had worse lung function and more serious dyspnea (both *P* < 0.05; Table 3, Supplementary Table S2).

**Table 3:** GOLD stages of COPD patients^†^.

**GOLD stages**	**Total**		**Low altitudes**		**High altitudes**		***P* ^a^**
	***n*** **/** ***N***	**Proportion (95%CI)**	***n*** **/** ***N***	**Proportion (95%CI)**	***n*** **/** ***N***	**Proportion (95%CI)**	
GOLD stage I	339/505	67.1% (63.0–71.5)	217/296	73.3% (68.0–78.2)	122/209	58.4% (50.7–64.9)	**0.001**
GOLD stage II	145/505	28.7% (24.8–33.1)	71/296	24.0% (19.2–29.1)	74/209	35.4% (28.9–42.2)	
GOLD stage III or IV	21/505	4.2% (2.6–5.9)	8/296	2.7% (1.0–4.6)	13/209	6.2% (3.1–9.9)	

GOLD, Global Initiative for Chronic Obstructive Lung Disease; COPD, chronic obstructive pulmonary disease; CI, confidence interval.

GOLD stage I (mild COPD): FEV_1_ ≥80% predicted; GOLD stage II (moderate COPD): FEV_1_ ≥50% to < 80% predicted; GOLD stage III (severe COPD): FEV_1_ ≥30% to < 50% predicted; GOLD stage IV (very severe): FEV_1_ < 30% predicted.

a Low altitudes vs. high altitudes.

† Three patients older than 81 years were excluded.

Bold values denote significant *P* values (*P* < 0.05).

### The role of high altitude on COPD

Biomass as fuel and ethnic minorities almost occurred at high altitudes (Table 1). Taking them into PSM would enormously decrease the sample size. In addition, the distributions of ethnicity and biomass as fuel between cases and controls were similar to the altitude, which might lead to multicollinearity and conceal the actual association between altitude and COPD if we take them into the PSM-processed and multivariate logistic regression models. Thus, the two variables were not considered in calculating propensity score (PS) and the multivariate logistic regression model.

**Figure 3:**
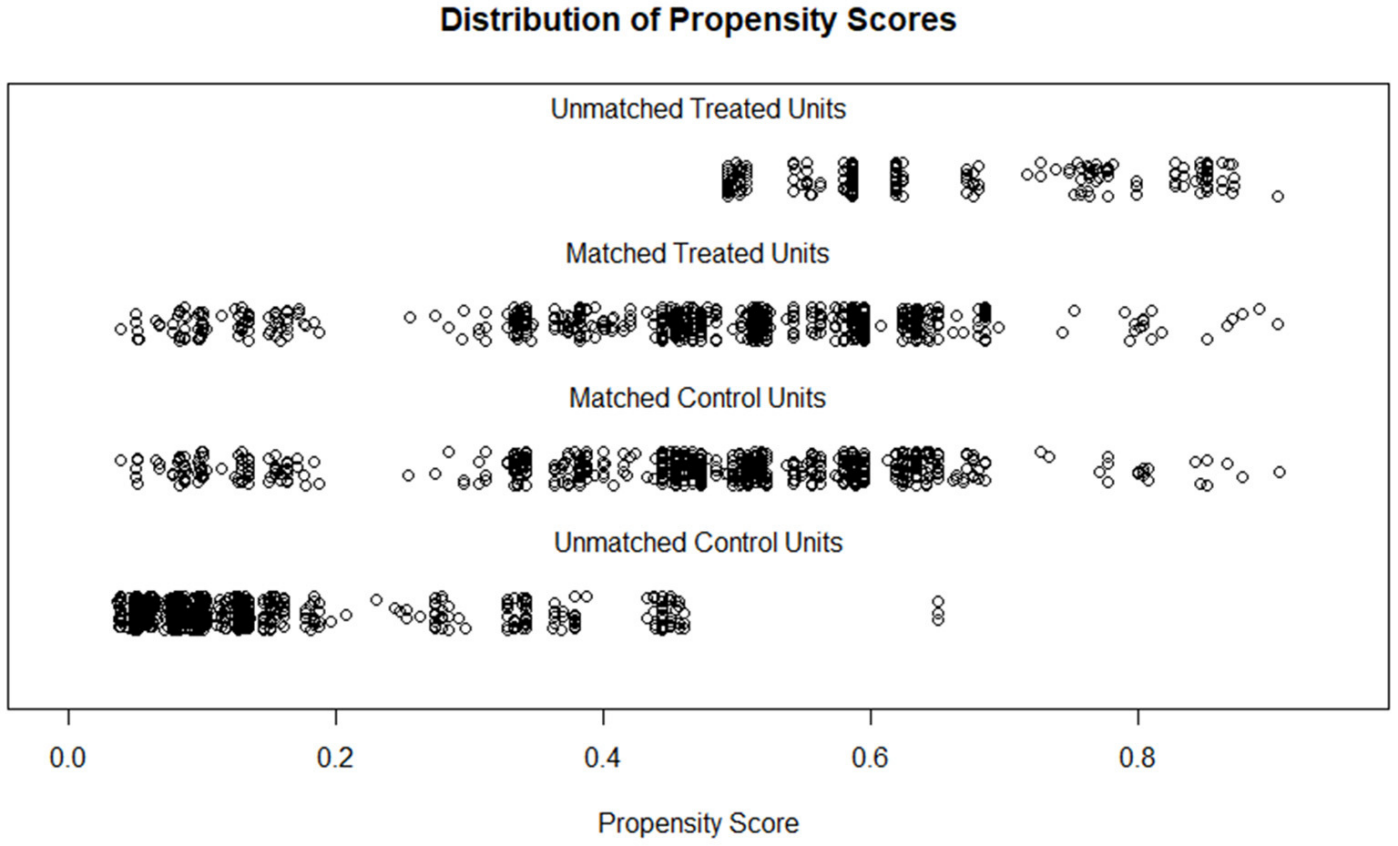
Distributions of propensity scores of participants before and after matching.

After matching, 699 participants were retained in each group; the two populations have similar distributions of PS and comparable characteristics (SMD < 0.2, *P* < 0.05; Figure 3, Supplementary Table S3). Further, logistic regression models indicated that high altitude increased COPD risk in both the criteria of FEV_1_/FVC < 0.7 [crude OR: 1.323 (1.084–1.615); Adjusted OR: 1.408 (1.107–1.79); PSM OR: 1.516 (1.162–1.978)] and FEV_1_/FVC < LLN [crude OR: 1.318 (1.084–1.602); adjusted OR: 1.544 (1.227–1.944); PSM OR: 1.599 (1.232–2.076); Table 4].

**Table 4:** Effect of altitude on the risk of COPD by logistic regression model.

**Variables**	**FEV**_**1**_**/FVC**<**0.7**			**FEV**_**1**_**/FVC**<**LLN**		
	**Crude OR**	**Adjusted OR** ^a^	**PSM OR** ^b^	**Crude OR**	**Adjusted OR** ^a^	**PSM OR** ^b^
Low altitudes	1.000 (ref.)	1.000 (ref.)	1.000 (ref.)	1.000 (ref.)	1.000 (ref.)	1.000 (ref.)
High altitudes	**1.323 (1.084–1.615)**	**1.408 (1.107–1.79)**	**1.516 (1.162–1.978)**	**1.318 (1.084–1.602)**	**1.544 (1.227–1.944)**	**1.599 (1.232–2.076)**

COPD, chronic obstructive pulmonary disease; OR, odd ratio; CI, confidence interval; PSM, propensity score matching.

a Adjusting age, sex, childhood hospital admission for severe respiratory disease, education level, coal as fuels, occupational exposure, smoking, BMI.

b Matching age, sex, childhood hospital admission for severe respiratory disease, education level, coal as fuels, occupational exposure, smoking, BMI.

Bold values denote significant *P* values (*P* < 0.05).

### Construction and validation of the nomogram

**Figure 4:**
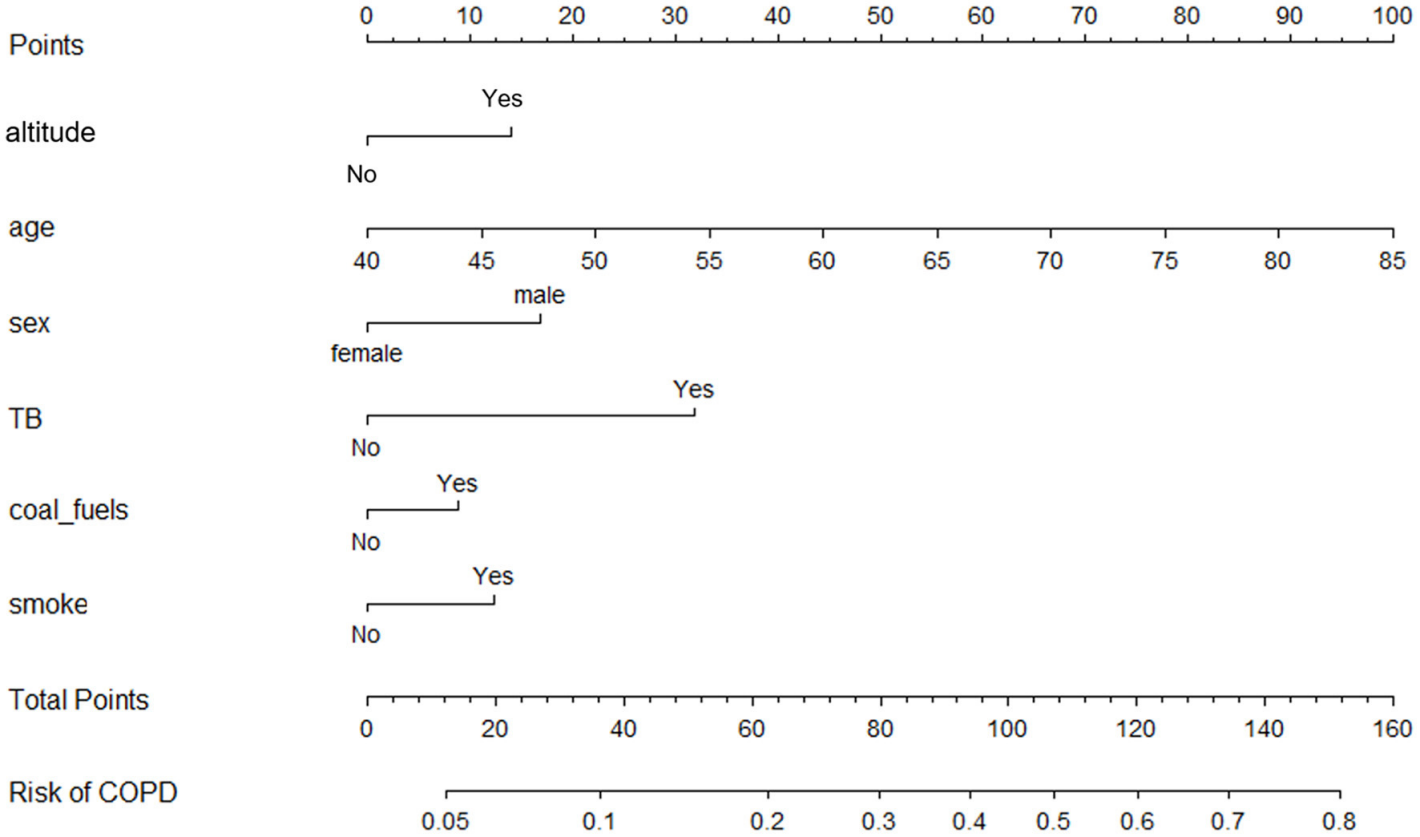
Nomogram for COPD reserving altitude, age, sex, history of tuberculosis, coal as fuel, and smoking status as predictors.

Participants were randomly divided into two sets. The training set had 1,491 individuals, while the validation set had 995 individuals. The difference in the characteristics between the two populations was not statistically significant (*P* > 0.05; Supplementary Table S4). The univariable logistic regression model first eliminated the factors that did not statistically associate with COPD. Then, the stepwise logistic regression model reserved altitude, age, sex, TB, coal as fuel, and smoking status for developing a nomogram (Table 5, Figure 4).

**Table 5:** Univariable and multivariable logistic regression analysis in training set.

**Variables**	**Cases/ controls**	**Univariable OR (95%CI)**	**Multivariable OR (95%CI)^a^**
Age (years)	308/1,183	**1.08 (1.06–1.09)**	1.07 (1.06–1.09)
**Sex**			
Female	99/594	Reference	Reference
Male	209/589	**2.13 (1.63–2.78)**	1.69 (1.18–2.41)
**Altitude**			
Low	172/785	Reference	Reference
High	136/398	**1.56 (1.21–2.01)**	1.55 (1.17–2.04)
**Childhood hospital admission for severe respiratory disease**			
No	284/1,115	Reference	–
Yes	24/68	1.39 (0.86–2.25)	–
**History of tuberculosis**			
No	302/1,177	Reference	Reference
Yes	6/6	**3.90 (1.25–12.17)**	2.68 (0.80–9.04)
**Educational level**			
Primary School or Below	184/652	Reference	–
Middle or high school	120/43	0.86 (0.67–1.12)	–
College or above	4/38	037 (0.13–1.06)	–
**Coal as fuel**			
No	127/561	Reference	Reference
Yes	181/622	**1.29 (1.00–1.66)**	1.32 (1.01–1.73)
**Biomass as fuel**			
No	46/197	Reference	–
Yes	262/986	1.14 (0.80–1.61)	–
**Smoking status**			
No	157/790	Reference	Reference
Yes	151/393	**1.93 (1.50–2.49)**	1.46 (1.03–2.08)
**Occupational exposure**			
No	196/756	Reference	–
Yes	112/457	0.91 (0.70–1.18)	–
**Ethnic groups**			
Han	215/929	Reference	–
Others	93/254	**1.58 (1.20–2.09)**	–
**BMI**			
Normal	180/632	Reference	–
Overweight	128/512	0.81 (0.61–1.07)	–
Obesity	51/202	0.84 (0.57–1.22)	–
malnutrition	9/26	1.55 (0.75–3.22)	–

BMI, body-mass index; OR, odd ratio; CI, confidence interval.

a Stepwise logistic regression model.

Bold values denote significant *P* values (*P* < 0.05).

**Figure 5:**
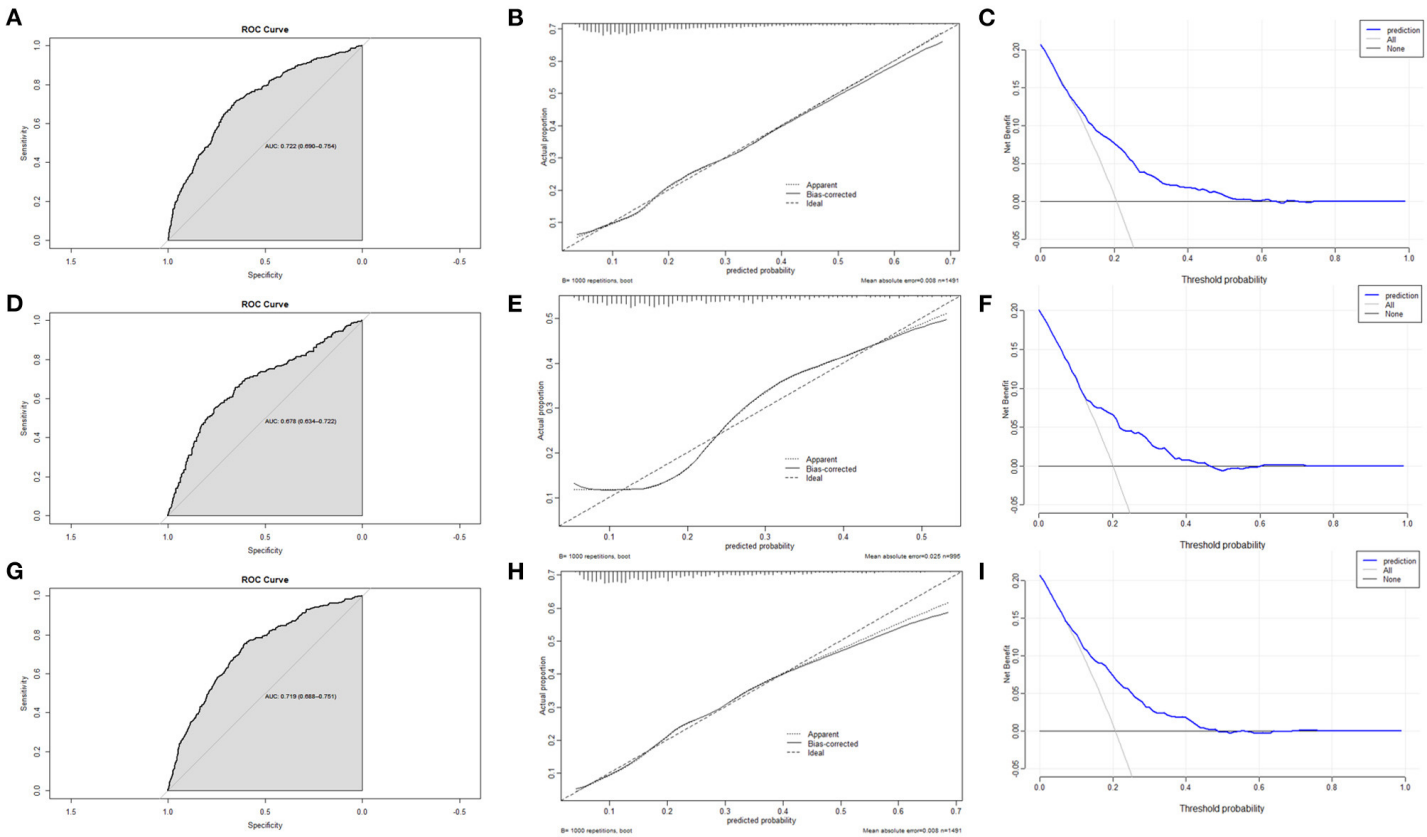
The assessment of the nomogram. **(A)** AUC of the ROC curve in training set was 0.722 (0.690–0.754). **(B)** Calibration curve in training set demonstrated that the predicted probabilities were almost identical to the actual probabilities. **(C)** Decision curve analysis in training set suggested that the clinical benefit was positive when the threshold probability ranged from 0.08 to 0.45. **(D)** AUC of the ROC curve in validation set was 0.678 (0.634–0.722). **(E)** Calibration curve in validation set demonstrated that the predicted probabilities fluctuated around the actual probabilities. **(F)** Decision curve analysis in validation set suggested that the clinical benefit was positive when the threshold probability ranged from 0.09 to 0.36. **(G)** Eliminating altitude, AUC of the ROC curve in training set was 0.719 (0.688–0.751). **(H)** Eliminating altitude, calibration curve in training set demonstrated that the predicted probabilities were more deviated from the actual probabilities. **(I)** Eliminating altitude, Decision curve analysis in training set suggested that the clinical benefit was positive when the threshold probability ranged from 0.07 to 0.37.

The ROC curve, calibration curve, and DCA were used to assess the efficiency of the nomogram. In the training set, the AUC was 0.722 (0.690–0.754); the predicted probabilities were almost identical to the actual probabilities; when the threshold probability ranged from 0.08 to 0.45, the clinical benefit was positive (Figures 5A–C). In the validation set, the AUC was 0.678 (0.634–0.722); the predicted probabilities also fluctuated around the actual probabilities; when the threshold probability ranged from 0.09 to 0.36, the clinical benefit was positive (Figures 5D–F). If removed altitude from the nomogram, the discrimination, calibration, and clinical benefit declined to some degree (Figures 5G–I).

## Discussion

In this cross-section study, we investigated a represented sample with 2,486 individuals aged 40 years or above in Gansu by the multistage stratified cluster sampling procedure. Using both the criteria of post-bronchodilator FEV1/FVC < 0.7 or LLN, we found that the prevalence and severity of COPD in high altitudes were higher than in low altitudes. Furthermore, the results of univariable, multivariable, and PSM-processed logistic regression models showed that high altitude was a risk factor for COPD. In addition, we identified altitude, age, sex, TB, coal as fuel, and smoking status as the risk factors and developed a nomogram for screening the high-risk population. The nomogram showed excellent discrimination, calibration, and clinical benefit in the internal validation.

**Figure 6:**
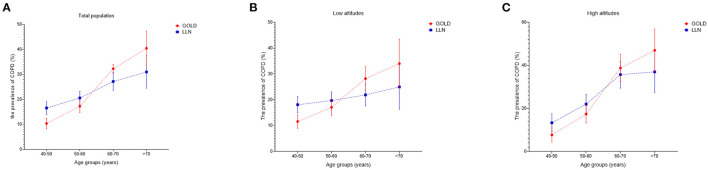
**(A–C)** Prevalence of COPD using LLN or GOLD criteria by age and altitude.

The prevalence of COPD in China has risen rapidly in recent years. Among residents older than 40, the overall prevalence was about 8.2% (7.9–8.6) in 2002–2004. However, that increased to 13.6% (12.0–15.2) just a decade later (3, 4). Fang et al. (4) invoked data from the surveillance points in northwest China, only two of which were located in Gansu, and concluded that the standardized prevalence in northwest China was 13.6% (9.5–17.6). However, after investigating four representative regions in Gansu, we found that the standardized prevalence in Gansu was 19.7% (17.9–21.6) diagnosed by the GOLD criterion, while that was 21.3% (19.5–23.3) diagnosed by post-bronchodilator FEV_1_/FVC < LLN. Previous studies found that unclean fuels were frequently used in northwest China (coal as fule: 49.4%; biomass as fule: 48.1%), but our study found a higher frequency in Gansu (coal as fule: 53.7%; biomass as fule: 83.9%), which might lead to the much higher prevalence in Gansu than in northwest China (4). Notedly, in the total, low-altitude and high-altitude populations, the prevalences diagnosed by post-bronchodilator FEV_1_/FVC < LLN were higher in the middle-aged and lower in the elderly when compared to the prevalences diagnosed by the GOLD criterion (Figures 6A–C). LLN as the diagnostic criterion might have an advantage in improving diagnostic sensitivity in the middle-aged and avoiding overdiagnosis in the elderly (22).

The role of altitude on COPD is still unclear (13). Some epidemiological studies indicated that high altitude was a protective factor for COPD (25–29). Recently, an epidemiological study combining several databases even suggested that high altitude (>1,500 m) did not associate with COPD (15). However, the participants of those studies were westerners whose genetic background might differ from Chinese (15, 25–29). In some research, the exposure frequencies of some known risk factors were lower at high altitudes, which contracted to our country (15, 25, 29). In addition, some studies either did not perform multivariate analysis or missed crucial risk factors in the model for adjusting confoundings (coal as fuel, etc.) (15, 26, 27, 29). We advocated that high altitude increased COPD risk and severity, which was also one of the mainstream views (14, 29–33). There were two reasonable explanations. On the one hand, high-altitude residents tended to use unclean energy for cooking and warming. Long-term exposure to indoor air pollutants might promote inflammation and oxidative stress in the lung (34). On the other hand, a climate like hypobaric and hypoxic would stimulate hypoxia-related genes, which might play a harmful role in a series of physiological and biochemical processes such as inflammation, lung development, pulmonary hypertension or remodeling, and vascular permeability (35, 36).

Our result only regarded age, sex, TB, coal as fuel, and smoking as the COPD-relative predictors. In the univariable analysis, childhood hospital admission for severe respiratory disease, educational level, biomass as fuel, occupational exposure, and BMI were not significantly associated with COPD. One of the reasons might be the crude evaluation of the exposures like biomass as fuel and occupational exposure. Besides, similar exposure frequencies of the above factors in cases and controls required a larger sample to identify the association between the risk factors and COPD (37). Noteworthily, ethnicity was significantly associated with COPD in the univariable analysis. But the stepwise logistic regression model eliminated ethnicity, which might cause by its strong association with altitude (38).

Based on the multivariable regression model, the nomogram provides a quick, visual, and accurate tool to predict the probability of clinical outcomes and is popular among medical workers (39). As we know, this tool is rarely utilized for screening the COPD high-risk population (40, 41). In this study, we creatively developed a nomogram for COPD screening, which was evaluated by the ROC curve, calibration curve, and DCA. In the training and validation sets, the nomogram demonstrated excellent calibration and clinical benefit. However, the AUC in the training set was 0.722 (0.690–0.754), while that in the validation set was 0.678 (0.634–0.722), merely indicating acceptable discrimination of the nomogram. More candidate predictors should be enrolled to improve the efficiency of the nomogram by expanding the sample size in future work. We also found that the discrimination, calibration, and clinical benefit declined after removing altitude from the nomogram, reiterating that altitude is an important risk factor for COPD.

This study has some limitations: (1) As a retrospective and cross-section study, the recall bias and defect in causal inference are inevitable. (2) The evaluation of some risk factors is crude, which may lead to false negative results. (3) The sample in high altitudes is relatively small, requiring a larger sample to detect the candidate risk factors with a similar distribution in cases and controls (37). (4) Our nomogram needs further external validation.

## Conclusions

COPD has become a severe public health problem in Gansu. High altitude is a critical risk factor besides aging, male, TB, coal as fuel, and smoking. The nomogram with the above risk factors has satisfactory efficiency in screening high-risk individuals.

## Data availability statement

The raw data supporting the conclusions of this article will be made available by the authors, without undue reservation.

## Ethics statement

The studies involving human participants were reviewed and approved by the Institutional Review Board of Guangzhou Medical University and Xi'an Jiaotong University Health Science Center—approval: GZMC2007-07-0676 and XJTU2016-411. The patients/participants provided their written informed consent to participate in this study.

## Author contributions

AL analyzed and interpreted the data and completed the writing. CM, BR, YW, GY, and HL took part in investigation. JL, XW, HZ, and XZ made contributions to conceptualization, participants recruitment, and project management. JL, YD, CX, and DH guided the methodology and revised the manuscript. All authors have read and approved the final manuscript.
