# Supplementary material for: Development and validation of nomogram including high altitude as a risk factor for COPD: A cross-sectional study based on Gansu population
Source: Front Public Health. 2023 Mar 2;11:1127566. doi: 10.3389/fpubh.2023.1127566 (PMC10019355; doi:10.3389/fpubh.2023.1127566)
Supplement: Supplementary file 1 [file Data_Sheet_1.docx]

**Table S1. Prevalence of COPD in Gansu participants aged 40 years and older by different characteristics (post-bronchodilator FEV_1_/FVC<LLN)** ^†^

| **Variables** | **Total** | |  | **Low altitudes** | |  | **High altitudes** | | ***P* ^a^** |
| --- | --- | --- | --- | --- | --- | --- | --- | --- | --- |
|  | **Case/total** | **Prevalence (95%CI)** |  | **Case/total** | **Prevalence (95%CI)** |  | **Case/total** | **Prevalence (95%CI)** |  |
| Overall | 538/2481 | 21.7% (20.2-23.3) |  | 316/1583 | 20.0% (18.0-22.1) |  | 222/898 | 24.7% (22.0-27.8) | **0.006** |
| Sex |  |  |  |  |  |  |  |  |  |
| Female | 192/1165 | 16.5% (14.5-18.4) |  | 92/682 | 13.5% (10.8-16.1) |  | 100/483 | 20.7% (17.2-24.4) | **0.001** |
| Male | 346/1316 | 26.3% (24.0-28.8) |  | 224/901 | 24.9% (22.1-27.6) |  | 122/415 | 29.4% (24.8-33.6) | 0.082 |
| *P* |  | **<0.001** |  |  | **<0.001** |  |  | **0.003** |  |
| Age (years) |  |  |  |  |  |  |  |  |  |
| 40-50 | 137/824 | 16.6% (14.0-19.4) |  | 104/576 | 18.1% (14.9-21.1) |  | 33/248 | 13.3 (8.9-17.7) | 0.093 |
| 50-60 | 179/870 | 20.6% (17.9-23.3) |  | 107/543 | 19.7% (16.5-23.2) |  | 72/327 | 22.0 (17.4-26.6) | 0.414 |
| 60-70 | 161/592 | 27.2% (23.6-30.5) |  | 80/365 | 21.9% (17.6-26.0) |  | 81/227 | 35.7 (29.8-42.1) | **<0.001** |
| ≥70 | 61/195 | 31.3% (24.6-38.0) |  | 25/99 | 25.3% (16.2-34.2) |  | 36/96 | 37.0 (27.6-47.4) | 0.065 |
| *P*_trend_ |  | **<0.001** |  |  | 0.051 |  |  | **<0.001** |  |
| Ethnic groups |  |  |  |  |  |  |  |  |  |
| Han | 398/1889 | 21.1% (19.2-23.0) |  | 315/1567 | 20.1% (18.1-22.2) |  | 83/322 | 25.8% (21.0-30.6) | **0.023** |
| Others | 140/592 | 23.6% (20.2-26.9) |  | 1/16 | 6.3% (0.0-20.0) |  | 139/576 | 24.1% (20.4-27.6) | 0.097 |
| *P* |  | 0.184 |  |  | 0.168 |  |  | 0.584 |  |
| Childhood hospital admission  for severe respiratory disease |  |  |  |  |  |  |  |  |  |
| No | 499/2323 | 21.5% (19.8-23.1) |  | 307/1531 | 20.1% (18.0-22.0) |  | 192/792 | 24.2% (21.0-27.3) | **0.020** |
| Yes | 39/158 | 24.7% (17.7-31.4) |  | 9/52 | 17.3% (8.0-27.9) |  | 30/106 | 28.3% (19.6-36.6) | 0.132 |
| *P* |  | 0.345 |  |  | 0.626 |  |  | 0.363 |  |
| History of tuberculosis |  |  |  |  |  |  |  |  |  |
| No | 531/2463 | 21.6% (19.9-23.3) |  | 315/1575 | 20.0% (17.9-22.0) |  | 216/888 | 24.3% (21.3-27.2) | **0.012** |
| Yes | 7/18 | 38.9% (15.8-62.5) |  | 1/8 | 12.5% (0.0-40.0) |  | 6/10 | 60.0% (25.0-90.9) | **0.040** |
| *P* |  | 0.075 |  |  | 0.597 |  |  | **0.009** |  |
| Educational level |  |  |  |  |  |  |  |  |  |
| Primary School or Below | 299/1392 | 21.5% (19.3-23.6) |  | 143/827 | 17.3% (14.7-19.9) |  | 156/565 | 27.6% (23.8-31.3) | **<0.001** |
| Middle or high school | 229/1018 | 22.5% (20.0-25.1) |  | 169/715 | 23.6% (20.6-26.9) |  | 60/303 | 19.8% (15.5-24.3) | 0.180 |
| College or above | 10/71 | 14.1% (6.7-22.6) |  | 4/41 | 9.8% (2.2-20.4) |  | 6/30 | 20.0% (7.7-36.4) | 0.220 |
| *P*_trend_ |  | 0.823 |  |  | **0.041** |  |  | **0.013** |  |
| Coal as fuel |  |  |  |  |  |  |  |  |  |
| No | 226/1147 | 19.7% (17.5-22.1) |  | 139/753 | 18.5% (15.8-21.1) |  | 87/394 | 22.1% (18.1-26.3) | 0.143 |
| Yes | 312/1334 | 23.4% (21.1-25.6) |  | 177/830 | 21.3% (18.3-24.2) |  | 135/504 | 26.8% (22.8-30.6) | **0.022** |
| *P* |  | **0.026** |  |  | 0.154 |  |  | 0.105 |  |
| Biomass as fuel |  |  |  |  |  |  |  |  |  |
| No | 84/399 | 21.1% (17.4-25.2) |  | 82/392 | 20.9% (17.1-25.1) |  | 2/7 | 28.6% (0.0-67.7) | 0.623 |
| Yes | 454/2082 | 21.8% (20.1-23.5) |  | 234/1191 | 19.6% (17.3-21.9) |  | 220/891 | 24.7% (21.7-27.6) | **0.006** |
| *P* |  | 0.738 |  |  | 0.585 |  |  | 0.813 |  |
| Smoking status |  |  |  |  |  |  |  |  |  |
| Never | 299/1584 | 18.9% (17.0-20.8) |  | 152/916 | 16.6% (13.9-19.2) |  | 147/668 | 22.0% (18.7-25.0) | **0.007** |
| Ever | 31/153 | 20.3% (14.3-26.5) |  | 23/129 | 17.8% (11.1-24.7) |  | 8/24 | 33.3% (15.0-54.5) | 0.083 |
| Now | 208/744 | 28.0% (25.0-30.9) |  | 141/538 | 26.2% (22.6-29.8) |  | 67/206 | 32.5% (26.3-39.0) | 0.086 |
| *P*_trend_ |  | **<0.001** |  |  | **<0.001** |  |  | **0.002** |  |
| Occupational exposure |  |  |  |  |  |  |  |  |  |
| No | 318/1511 | 21.0% (19.0-23.0) |  | 128/714 | 17.9% (15.3-20.8) |  | 190/797 | 23.8% (20.8-27.0) | **0.005** |
| Yes | 220/970 | 22.7% (20.1-25.4) |  | 188/869 | 21.6% (18.8-24.4) |  | 32/101 | 31.7% (22.5-41.2) | **0.022** |
| *P* |  | 0.335 |  |  | 0.066 |  |  | 0.085 |  |
| BMI |  |  |  |  |  |  |  |  |  |
| Normal | 256/1163 | 22.0% (19.6-24.3) |  | 145/733 | 19.8% (16.8-22.6) |  | 111/430 | 25.8% (21.5-30.1) | **0.019** |
| Overweight | 186/902 | 20.6% (18.0-23.5) |  | 112/575 | 19.5% (16.0-22.8) |  | 74/327 | 22.6% (17.9-27.0) | 0.261 |
| Obesity | 82/364 | 22.5% (18.2-26.8) |  | 52/246 | 21.1% (16.2-26.3) |  | 30/118 | 25.4% (17.7-33.3) | 0.360 |
| malnutrition | 14/52 | 26.9% (14.8-39.5) |  | 7/29 | 24.1% (8.7-40.7) |  | 7/23 | 30.4% (12.5-52.2) | 0.611 |
| *P*_trend_ |  | 0.771 |  |  | 0.596 |  |  | 0.903 |  |
| COPD, chronic obstructive pulmonary disease; BMI, body-mass index; CI, confidence interval. | | | | | | | | | |
| ^a^ Low altitudes vs high altitudes  ^†^ 5 patients older than 81 years were excluded. | | | | | | | | | |

**Table S2. Modified MRC** **dyspnea scale for COPD patients [%(95%CI)]**

| **Groups** | **Total** | |  | **Low altitudes** | |  | **High altitudes** | | ***P ^a^*** |
| --- | --- | --- | --- | --- | --- | --- | --- | --- | --- |
|  | **Case** | **Proportion** |  | **Case** | **Proportion** |  | **Case** | **Proportion** |  |
| Total | 508 |  |  | 297 |  |  | 211 |  |  |
| mMRC |  |  |  |  |  |  |  |  | **<0.001** |
| Grade 0 | 280 | 55.1% (51.0-59.3) |  | 202 | 68.0% (63.0-73.4) |  | 78 | 37.0% (30.8-43.6) |  |
| Grade 1 | 88 | 17.3% (14.0-20.7) |  | 42 | 14.1% (10.4-18.5) |  | 46 | 21.8% (16.1-27.5) |  |
| Grade 2 | 86 | 16.7% (13.8-20.3) |  | 47 | 15.8% (11.8-19.5) |  | 39 | 18.5% (13.7-23.7) |  |
| Grade 3 | 27 | 5.3% (3.31-7.3) |  | 4 | 1.3% (0.0, 2.7) |  | 23 | 10.9% (6.6-15.2) |  |
| Grade 4 | 27 | 5.3% (3.31-7.3) |  | 2 | 0.7% (0.0, 1.7) |  | 25 | 11.8% (7.6-16.1) |  |
| mMRC, modified Medical Research Council; COPD, chronic obstructive pulmonary disease; CI, confidence interva.  ^a^ Low altitudes vs high altitudes. | | | | | | | | | |

**Table S3. Heterogeneity of characteristics between two groups after propensity score matching**

| **Variables** | **Low altitudes (n=699)** | **High altitudes (n=699)** | ***P*** | **SMD** |
| --- | --- | --- | --- | --- |
| Age (years) |  |  |  |  |
| ＜60 | 482 (69.0%) | 477 (68.2%) | 0.818 | 0.015 |
| ≥60 | 217 (31.0%) | 222 (31.8%) |  |  |
| Sex |  |  |  |  |
| Female | 342 (48.9%) | 355 (50.8%) | 0.521 | 0.037 |
| Male | 357 (51.1%) | 344 (49.2%) |  |  |
| Childhood hospital admission  for severe respiratory disease |  |  |  |  |
| No | 662 (94.7%) | 662 (94.7%) | 1.000 | <0.001 |
| Yes | 37 (5.3%) | 37 (5.3%) |  |  |
| History of tuberculosis |  |  |  |  |
| No | 695 (99.4%) | 692 (99.0%) | 0.545 | 0.049 |
| Yes | 4 (0.6%) | 7 (1.0%) |  |  |
| Educational level |  |  |  |  |
| Primary School or Below | 417 (59.7%) | 434 (62.1%) | 0.513 | 0.062 |
| Middle or high school | 261 (37.3%) | 241 (34.5%) |  |  |
| College or above | 21 (3.0%) | 24 (3.4%) |  |  |
| Coal as fuel |  |  |  |  |
| No | 292 (41.8%) | 289 (41.3%) | 0.914 | 0.009 |
| Yes | 407 (58.2%) | 410 (58.7%) |  |  |
| Smoking status |  |  |  |  |
| No | 480 (68.7%) | 492 (70.4%) | 0.523 | 0.037 |
| Yes | 219 (31.3%) | 207 (29.6%) |  |  |
| Occupational exposure |  |  |  |  |
| No | 600 (85.8%) | 598 (85.6%) | 0.939 | 0.008 |
| Yes | 99 (14.2%) | 101 (14.4%) |  |  |
| BMI |  |  |  |  |
| Normal | 338 (48.4%) | 362 (51.8%) | 0.236 | 0.110 |
| Overweight | 247 (35.3%) | 215 (30.8%) |  |  |
| Obesity | 96 (13.7%) | 108 (15.5%) |  |  |
| malnutrition | 18 (2.6%) | 14 (2.0%) |  |  |
| BMI, body-mass index; SMD, standardized mean difference. | | | | |

**Table S4. Demographic characteristics and exposures of the training set and validation set**

| **Variables** | **Training set (n=1491)** | **Validation set (n=995)** | ***P ^d^*** |
| --- | --- | --- | --- |
| Age (years) ^a^ | 55.30±9.39 | 54.38±9.23 | 0.349 |
| BMI (kg/m²) ^a^ | 24.32±3.55 | 24.46±3.37 | 0.236 |
| Sex |  |  |  |
| Female | 693 (46.5%) | 473 (47.5%) | 0.604 |
| Male | 798 (53.5%) | 522 (52.5%) |  |
| Ethnic groups |  |  |  |
| Han | 1144 (76.7%) | 746 (75.0%) | 0.316 |
| Others | 347 (23.3%) | 249 (25.0%) |  |
| Altitude |  |  |  |
| Low | 957 (64.2%) | 627 (63.0%) | 0.552 |
| High | 534 (35.8%) | 368 (37.0%) |  |
| Childhood hospital admission for  severe respiratory disease |  |  |  |
| No | 1399 (93.8%) | 928 (93.3%) | 0.574 |
| Yes | 92 (6.2%) | 67 (6.7%) |  |
| History of tuberculosis |  |  |  |
| No | 1479 (99.2%) | 989 (99.4%) | 0.561 |
| Yes | 12 (0.8%) | 6 (0.6%) |  |
| Educational level |  |  |  |
| Primary School or Below | 836 (56.1%) | 558 (56.1%) | 0.989 |
| Middle or high school | 613 (41.1%) | 408 (41.0%) |  |
| College or above | 42 (2.8%) | 29 (2.9%) |  |
| Coal as fuel |  |  |  |
| No | 688 (46.1%) | 463 (46.5%) | 0.849 |
| Yes | 803 (53.9%) | 532 (53.5%) |  |
| Biomass as fuel |  |  |  |
| No | 243 (16.3%) | 157 (15.8%) | 0.730 |
| Yes | 1248 (83.7%) | 838 (84.2%) |  |
| Smoking status |  |  |  |
| No | 947 (63.5%) | 641 (64.4%) | 0.644 |
| Yes | 544 (36.5%) | 354 (35.6%) |  |
| Occupational exposure |  |  |  |
| No | 922 (61.8%) | 594 (59.7%) | 0.284 |
| Yes | 569 (38.2%) | 401 (40.3%) |  |
| Pre-FEV_1_ (L) ^b^ | 2.60 (2.20, 3.20) | 2.60 (2.20, 3.10) | 0.325 |
| Pre-FVC (L) ^b^ | 3.50 (2.90, 4.30) | 3.50 (3.00, 4.30) | 0.381 |
| Pre-FEV_1_/FVC (%) ^b^ | 79.50 (69.00, 79.50) | 75.2 (69.00, 79.80) | 0.461 |
| BMI, body-mass index; FVC, forced vital capacity; FEV1, forced expiratory volume in the first second.  ^a^ mean (standard deviation, SD).  ^b^ median (P_25_, P_75_).  ^d^ Training set vs validation set. | | | |
